# Novel AAV-Based Rat Model of Forebrain Synucleinopathy Shows Extensive Pathologies and Progressive Loss of Cholinergic Interneurons

**DOI:** 10.1371/journal.pone.0100869

**Published:** 2014-07-07

**Authors:** Patrick Aldrin-Kirk, Marcus Davidsson, Staffan Holmqvist, Jia-Yi Li, Tomas Björklund

**Affiliations:** 1 Molecular Neuromodulation, Wallenberg Neuroscience Center, Lund University, Lund, Sweden; 2 Neuronal Plasticity and Repair, Wallenberg Neuroscience Center, Lund University, Lund, Sweden; UCL Institute of Neurology, United Kingdom

## Abstract

Synucleinopathies, characterized by intracellular aggregation of α-synuclein protein, share a number of features in pathology and disease progression. However, the vulnerable cell population differs significantly between the disorders, despite being caused by the same protein. While the vulnerability of dopamine cells in the substantia nigra to α-synuclein over-expression, and its link to Parkinson's disease, is well studied, animal models recapitulating the cortical degeneration in dementia with Lewy-bodies (DLB) are much less mature. The aim of this study was to develop a first rat model of widespread progressive synucleinopathy throughout the forebrain using adeno-associated viral (AAV) vector mediated gene delivery. Through bilateral injection of an AAV6 vector expressing human wild-type α-synuclein into the forebrain of neonatal rats, we were able to achieve widespread, robust α-synuclein expression with preferential expression in the frontal cortex. These animals displayed a progressive emergence of hyper-locomotion and dysregulated response to the dopaminergic agonist apomorphine. The animals receiving the α-synuclein vector displayed significant α-synuclein pathology including intra-cellular inclusion bodies, axonal pathology and elevated levels of phosphorylated α-synuclein, accompanied by significant loss of cortical neurons and a progressive reduction in both cortical and striatal ChAT positive interneurons. Furthermore, we found evidence of α-synuclein sequestered by IBA-1 positive microglia, which was coupled with a distinct change in morphology. In areas of most prominent pathology, the total α-synuclein levels were increased to, on average, two-fold, which is similar to the levels observed in patients with SNCA gene triplication, associated with cortical Lewy body pathology. This study provides a novel rat model of progressive cortical synucleinopathy, showing for the first time that cholinergic interneurons are vulnerable to α-synuclein over-expression. This animal model provides a powerful new tool for studies of neuronal degeneration in conditions of widespread cortical α-synuclein pathology, such as DLB, as well an attractive model for the exploration of novel biomarkers.

## Introduction

Synucleinopathies such as Parkinson's disease (PD) and dementia with Lewy bodies (DLB) are neurological disorders, that while differing significantly in their symptomatic presentation, are unified by α-synuclein aggregation and neuronal degeneration [1], [2]. Between these two distinct diagnoses lies also a spectrum of disorders usually classified as PD with dementia (PDD) [3]. DLB is one of the more common forms of dementia although the exact proportion of all the dementia patients that is actually DLB (as determined through neuropathological examination) is still not settled. Studies indicate however, that DLB and vascular origins of dementia are a close tie for the second most common source of dementia after Alzheimer's disease (AD) [4]. One of the striking differentiators of DLB is thought to be caused by α-synuclein toxicity localized to the cerebral cortices, leading to neurodegeneration and in particular, loss of cortical acetylcholine [5],[6]. Together this causes frontal-subcortical and visio-spatial dysfunction, manifested by rapidly progressing dementia, apathy, depression and vivid visual hallucinations. Memory impairment may or may not be part of the initial symptoms however. Through insertion of the human α-synuclein gene in mice using transgenic techniques, a number of useful animal models have been created replicating α-synuclein pathology in PD [7]–[11]. Other useful animal models have been created through targeted gene delivery using viral vectors into the midbrain dopaminergic neurons of mice, rats and non-human primates [12]–[16] or the combination of both transgenics and viral vectors [17]. Transgenic animal models have been important for understanding the neuronal pathology and motor dysfunction seen in PD but lack the robust cortical pathology, replicating the cognitive dysfunction seen in DLB. Likewise, targeted gene delivery has been used mainly to over-express α-synuclein in small, well defined brain regions like the substantia nigra pars compacta (SNpc), replicating primarily motor symptoms of PD.

Targeting a large brain region, such as the cerebral cortices, using recombinant viral vector mediated gene transfer has, however, remained a challenge. To overcome the limitation in viral vector diffusion in the adult brain parenchyma, a novel strategy was proposed to inject the viral vector suspension into the maturing brain during the first post-natal days [10], [18], [19]. This mode of administration enables the vector diffusion and infection to occur before maturation of axon myelination and gliogenesis, the two processes that restrict free diffusion in the brain. Using this methodology to deliver human α-synuclein in a recombinant adeno-associated viral (AAV) serotype 6 vector (AAV6), we were able to create a novel rat model exhibiting α-synuclein over-expression mainly localized to the cerebral cortices, mimicking primarily cerebral type of Lewy body disease [20]. These animals displayed a progressive neurodegenerative phenotype with both widespread degeneration of cholinergic interneurons and a multitude of degenerative changes in the remaining cortical neurons. This was associated with a changed behavioral phenotype displaying increased baseline locomotor activity as well as dopaminergic dysregulation.

## Materials and Methods

### Experimental procedure

To evaluate the significance of α-synuclein over-expression localized to the forebrain, neonatal Sprague Dawley rats received under hypothermia a stereotactic, intra-parenchymal injection of an AAV6 vector containing constructs expressing either α-synuclein (n = 29) or GFP (n = 21). A third group, exposed only to the hypothermic treatment, was kept as intact controls (n = 22). All animals were evaluated for deficits in memory function in Morris water maze; general anxiety; responsiveness of the dopaminergic motor system and general motor function, with a subgroup being tested at 8 weeks and the remainder tested at 6 months. Animals were sacrificed ten months post viral-injection and evaluated for cortical neuronal degeneration using a modified isotropic fractionation protocol [21]. In addition, further *post mortem* analyses were performed using immunohistochemistry.

### Animal Research

Six time-mated, pregnant female rats were purchased from Charles River (Germany). After birth of the litter they were housed one litter per cage with free access to food and water under a 12 hours light/12 hours dark cycle in a temperature-controlled room. All experimental procedures performed in this study were approved by the Ethical Committee for Use of Laboratory Animals in the Lund-Malmö region.

### Neonatal stereotaxic surgery

We used a neonatal model where pups were injected post-natal day 2–4, in order to facilitate neuronal transduction throughout the forebrain. Rat pups were anesthetized by hypothermia through the following procedure; The pups were individually sedated through rapid cooling in wet ice for 5 minutes where after the animal was retained at ≈+4°C on a metal bed in the stereotax throughout the surgery. A small hole was drilled through the skull and the viral vector solution (viral titer: 3.5E12 gc/ml) was injected bilaterally (1 µl/side) into the striatum at a flow rate of 0.2 µl/min using a pulled glass capillary (60–80 µm I.D. and 120–160 µm O.D.) attached to a 5 µl hamilton syringe. Target sites for the injection were identified by using coordinates (relative to the bregma): AP = +0.7; ML = +2.1; DV = −2.9 and the capillary was left in position for one minute before retraction. Post-surgery, the wound was closed using a surgical suture and the animal was placed on a heating mat until awake.

### Recombinant AAV vectors

AAV vector plasmids containing genes for either wild type human α-synuclein or GFP, were expressed under the human synapsin-1 promoter, ensuring neuron specific protein expression [22]. The sequence was flanked 3′ by the Woodchuck hepatitis virus post-transcriptional regulatory element (WPRE) and terminated with a SV40 derived poly-adenylation sequences to increase mRNA cytosolic half-life. AAV vector constructs were produced by dual-plasmid, calcium precipitation mediated transient transfection of HEK-293 cells and purified by iodixanol gradient centrifugation and anion exchange chromatography according to [23]. Viral titers were determined by qPCR with primers recognizing the WPRE sequence and diluted to match the working titer of 3.5E12 GC/ml [24].

### Evaluation of learning and memory impairments

Learning and memory deficits, due to α-synuclein over-expression and toxicity, were evaluated in the Morris water maze (MWM) task at six months post-injection. The MWM test was performed as described in [25]. In brief, the rats learning and global visio-spatial working memory abilities were assessed by tracking the mean time for each animal to find a platform submerged in a 2 m I.D. water filled tank, over a period of seven days, 4 trials/day. Retention of the performance of the task was assessed by additional trials in the MWM at one, three, seven and 21 days after the initial learning phase. Time spent searching for the platform and total distance traveled was recorded using the Ethovision 2.0 tracking software.

### General motor and exploratory behavior

General motor function and exploratory behavior in animals expressing α-synuclein was assessed by open field experiment at six months post viral injection. Animals were placed in the open field boxes (40×40×40 cm) and their, general mobility, rearing and location was recorded over 1 hour by use of infrared sensors and tracked by the San Diego Instruments tracking software. Activity was recorded in a dark environment.

### Assessment of dopaminergic pathways

Deficits in dopaminergic neurotransmission were evaluated in the open field boxes following subcutaneous apomorphine injection (1 mg/kg s.c.), at nine months post-injection. The locomotor response to dopamine receptor activation by apomorphine was recorded over 1 hour, in the open field boxes, as above.

### Tissue preparation

At 8 weeks post injection a subgroup of the animals were killed (AAV6|a-Syn n = 4, AAV6|GFP, n = 6 Intact ctrl n = 8). At 10 months post AAV injection, all but seven of the remaining (AAV6|a-Syn n = 22, AAV6|GFP n = 13 Intact ctrl n = 12) were killed. The animals were deeply anesthetized by sodium pentobarbital overdose (Apoteksbolaget, Sweden) and transcardially perfused with 50 ml physiological saline solution followed by 250 ml of freshly prepared, ice-cold, 4% paraformaldehyde (PFA) in 0.1 M phosphate buffer adjusted to pH = 7.4. The brains were then removed and fixed further for 2 hours in cold PFA before storing in 25% buffered sucrose for cryoprotection over at least 24 hours until further processing. The remaining seven animals were killed by decapitation, where after the brain was removed and sliced in the coronal axis into two millimeter thick slices using a brain mold. The striatal tissue, midbrain region, olfactory bulb and two regions of the cerebral cortex (covering frontal or motor cortex) were rapidly dissected and frozen individually on dry ice and stored at −80°C until further analysis with western blot and qPCR.

### Neuronal quantification through isotropic fractionation

PFA-fixed complete cerebral cortex was dissected bilaterally from AAV6|a-Syn (n = 6), AAV6|GFP (n = 5) and intact control rats (n = 6), killed 10 months after viral injection. The protocol used here is a modified version of that published by Herculano-Houzel and collaborators [21]. The tissue was dissociated in 10 ml of 0.5 M tri-sodium citrate buffer (pH 4.5) into homogenous nuclei solutions using the GentleMacs dissociator (Miltenyi biotec). Heating the sample between dissociation programs further facilitated dissociation, allowing for a homogeneous nuclei suspension. The nuclei solution was subjected to antigen retrieval in 0.2 M boric acid (pH 9.0) at 70°C for one hour, followed by blocking in 5% BSA for one hour. DAPI was added to stain all nuclei, allowing for quantification of neuronal and non-neuronal cells. In order to differentiate neuronal nuclei from non-neuronal nuclei within the same sample, the nuclei were stained using a neuronal specific monoclonal mouse anti-NeuN antibody (1∶200; MAB377 Millipore) in conjunction with a Dylight 550 goat, anti-mouse secondary antibody (1∶400; ab96880, Abcam). Samples from the nuclei solution were then counted in a haemocytometer using fluorescence-microscopy to calculate the concentration of nuclei. In order to validate our modified protocol, the cerebellum of the animals were also dissociated and quantified using the same modified protocol.

### Immunohistochemistry

The remaining PFA fixed brains were cut into 35 µm thick coronal sections using a freezing microtome (Leica SM2000R) and collected into 8 series and stored in anti-freeze solution (0.5M sodium phosphate buffer, 30% glycerol and 30% ethylene glycol) at −20°C until further processing.

Brain sections were evaluated for; human α-synuclein, rat α-synuclein and GFP expression patterns, using the mouse 211 anti α-synuclein antibody (1∶2000; sc-12767, Santa Cruz), mouse anti 42/α-synuclein (1∶1000; 610786, BD Biosciences) and chicken anti GFP (1∶20000; ab13970, Abcam) were used respectively. Presence of phosphorylated α-synuclein using the Rabbit anti-Serine129-phosphorylated α-synuclein (1∶2000; ab59264, Abcam). In order to evaluate the impact of α-synuclein over-expression on cholinergic neurons, midbrain dopaminergic neurons, astrocytes and microglia the goat anti-Choline acetyl transferase (1∶500; AB144P, Millipore), mouse anti-TH (1∶10000; 22941, Immunostar) Rabbit anti-GFAP (1∶1000; ab7260, Abcam) and Rabbit anti-IBA-1 (1∶2000; 019-19741, Wako) were used respectively. In addition, presence of phosphorylated Tau protein was assessed using the rabbit anti-tau phospho S396 antibody (1∶1000; ab32057 Abcam) Primary antibodies were visualized using biotinylated secondary antibodies: horse anti mouse (1∶250; BA2000, Vector Laboratories), horse anti goat (1∶250; BA9500, Vector laboratories), goat anti rabbit (1∶250; BA1000, Vector laboratories) and goat anti chicken (1∶250; BA9010, Vector laboratories). This was followed by a 30 minute incubation with avidin-biotin peroxidase solution (ABC Elite, Vector Laboratories) and developed by 3, 3′-diaminobenzidine (DAB) in 0.01% H_2_O_2_ color reaction. For fluorescence microscopy analysis, secondary antibodies used included: Dylight 550, goat anti mouse (1∶250; ab96880, Abcam), Dylight 488 goat anti mouse (1∶250; ab96871) Alexa 488, goat anti chicken (1∶250; A11039, Life technologies), Alexa 488 goat anti rabbit (1∶250; A11008, Life technologies) and Alexa 546 donkey anti goat (1∶250; A11056 Life technologies). All secondary antibodies were incubated for two hours in TBS-buffer.

### Laser scanning confocal microscopy

Laser scanning confocal microscopy was conducted using either a Leica SP2 or a Leica SP8 microscope. The confocal images were captured using a HyD detector. All images were acquired with the lasers activated in sequential mode, to avoid any bleed-through of fluorescence. Solid state lasers at wavelengths 488 nm and 552 nm were utilized to excite the respective fluorophores. The pinhole was retained at Airy 1 for all acquisitions. For each acquisition at the same magnification, identical settings were loaded for laser power gain etc. Post acquisition, deconvolution was performed using the “Deconvolution” plugin for ImageJ (developed by the Biomedical Imaging Group [BIG] - EPFL – Switzerland http://bigwww.epfl.ch/) utilizing the Richardson-Lucy algorithm and applying point-spreads functions (PSFs) calculated for the specific imaging equipment using the Gibson and Lanni model in the PSF Generator (BIG, EPFL – Switzerland http://bigwww.epfl.ch/algorithms/psfgenerator/). The same PFS models and deconvolution parameters were applied to all image stacks at the same magnification. Orthogonal or maximum intensity projections were generated using ImageJ64 (version 1.47).

### Western blot analysis

In order to evaluate the expression levels of α-synuclein protein, quantitative western blotting was used. Protein was extracted from dissected olfactory bulb, frontal cortex, motor cortex and striatum from AAV6|a-Syn (n = 3) AAV6|GFP (n = 2) and Intact ctrl (n = 2) groups. The dissected tissue was homogenized in Trizol, using the Fastprep-24 homogenizer (#116004500, MP Biomedicals) with lysing matrix D (#116913500, MP Biomedicals) Protein was then extracted and purified, using the Invitrogen trizol protein isolation protocol. Total extracted protein was quantified using the Pierce BCA protein assay kit (#23227, Thermo scientific). For total α-synuclein quantification, 20 µg of protein was loaded from each sample into a 12 well mini-protean TGX gel (#456-1095; 4–20%, 20 µl, BioRad) together with a standard of monomeric α-synuclein (50, 100, 200 ng). The molecular weight of the protein bands was determined using the Pageruler plus pre-stained protein ladder 10–250 kDA (#26619, Thermo scientific) and run at 200 volts for 40 minutes. Proteins were transferred to a PVDF membrane (0,2 µm) using the Transblot turbo transfer system (BioRad). Following a washing step, the membrane was blocked using 2% milk and incubated with the mouse anti 42/α-synuclein (1∶1000; 610786, BD Biosciences) over night in RT. Secondary HRP-conjugated goat anti mouse (1∶10000; 115-035-174, Jackson ImmunoResearch) were used and incubated in 2% milk for 2 hours. The membrane was then visualized using the Immun-Star HRP Chemiluminescent Substrate Kit (#170-5040, BioRad) with fluorescence detected using the Chemidoc MP imaging system (BioRad). Total α-synuclein was then quantified relative to the human α-synuclein monomeric standards.

### Quantitative PCR

Brain areas were carefully dissected and swiftly flash frozen using dry ice. mRNA was then extracted using Trizol (Invitrogen) followed by Aurum Total RNA Mini Kit (BioRad). 500 ng total mRNA was used in a cDNA synthesis using iScript cDNA Synthesis Kit (BioRad) with the following program; 25°C for 5 minutes, 42°C for 30 minutes and finally 85°C for 5 minutes.

Specific primer pairs for rat α-synuclein (fwd 5′-GCTGGGAACATTGCTGCT-3′, rev 5′-TGGGTACCCTTCTTCACCC-3′) and human α-synuclein (fwd 5′- CAGGGAGCATTGCAGCA3′, rev 5′-GTGGGGCTCCTTCTTCATTC-3′) were designed and validated on plasmid cDNA containing either rat or human α-synuclein. Both primer pairs were used on both cDNA and a fold change of 47000 was observed when using rat specific primers on human cDNA and 112500 fold change when using human specific primers on rat cDNA. Hence the primers were considered to be species specific. Prior to qPCR, primer efficiency was determined by qPCR on a 5 step dilution series of template, primer efficiencies were calculated and values were used in normalization.

All qPCR results were normalized to three reference genes, ActB (fwd 5′- AAGTCCCTCACCCTCCCAAAAG-3′, rev 5′-AAGCAATGCTGTCACCTTCCC-3′) GAPDH (fwd 5′-CAACTCCCTCAAGATTGTCAGCAA-3′, rev 5′-GGCATGGACTGTGGTCATGA-3′) and TBP (fwd 5′-TGGGATTGTACCACAGCTCCA-3′, rev 5′-CTCATGATGACTGCAGCAAACC-3′). All plates were run with inter-run calibrators and –RT controls.

Each reaction contained 10 µl SSoAdvanced SYBR Green Supermix (BioRad), 0.5 µM of each primer, 1 µl cDNA and 8 µl H_2_0. All samples were run in triplicates with the following program; 95°C for 30 sec, 95° for 5 sec and 60°C for 20 sec (39 cycles), followed by a melt curve ranging from 65°C to 95°C with 0.5°C increments every fifth second.

### Transmission electron microscopy

Tissue for TEM analysis was fixed using 1.5% paraformaldehyde with 1.5% glutaraldehyde in 0.1 M Sorensen's buffer for osmium tetroxide staining. The tissue was then fixed in 1% osmium tetroxide in 0.1 M Sorensen's buffer and progressively dehydrated in acetone, starting at 30% up to 100% acetone. The tissue was then embedded using 1∶1 acetone and epoxy and counterstained using 4% uranyl acetate and 1% lead citrate. Tissue used for immunogold staining was fixed using 1.5% paraformaldehyde with 0.5% glutaraldehyde and progressively dehydrated in ethanol, using the Leica AFS protocol and then embedded in Lowicryl HM20. The tissue was sectioned using the Leica UC7 ultramicrotome and sectioned into 50–60 nm slices. For immunostaining, gold conjugated syn 211 antibodies (santa-cruz 1∶200) were used and counterstained using 4% uranyl actetate. All tissue sections were analyzed using the Tecnai biotwin 120 KV microscope.

### Statistical analyses

Statistical analysis was performed using the SPSS 21 software. Datasets from the open field task, total neuronal quantification and quantification of ChAT positive neurons were tested for statistical significance using one-way ANOVA with Newman-Keuls post hoc test for multiple comparisons. Datasets for total human α-synuclein mRNA expression and total protein levels were tested for statistical significance using the Mann-Whitney rank order test.

## Results

In preparation for the described study, we compared AAV serotypes 5 and 6 in the neonatal rat transduction model. We found that the use of the AAV6 serotype ensured a broad spread of expression, mainly localized to the cerebral cortices while the AAV5 serotype, while displaying a broad spread, did not show a great affinity to the cerebral cortices. Stereotactic surgery on neonatal animals has in the past presented with its own challenges. However, survival rate of over 80% was achieved in our study by inducing anesthesia through hypothermia on neonatal (post-natal day 2–4) animals. In total, this resulted in 29 animals with a broad α-synuclein over-expression, localized to the cerebral cortices. To characterize disease progression, neuronal degeneration and pathology, the animals were exposed to a battery of behavior tests, assessing both motor and cognitive function, at either 8 weeks or 26 weeks post viral injections.

### AAV6|a-Syn animals display increased exploratory motor behavior and dysregulated response to dopamine agonist, but no memory impairment

**Figure 1:**
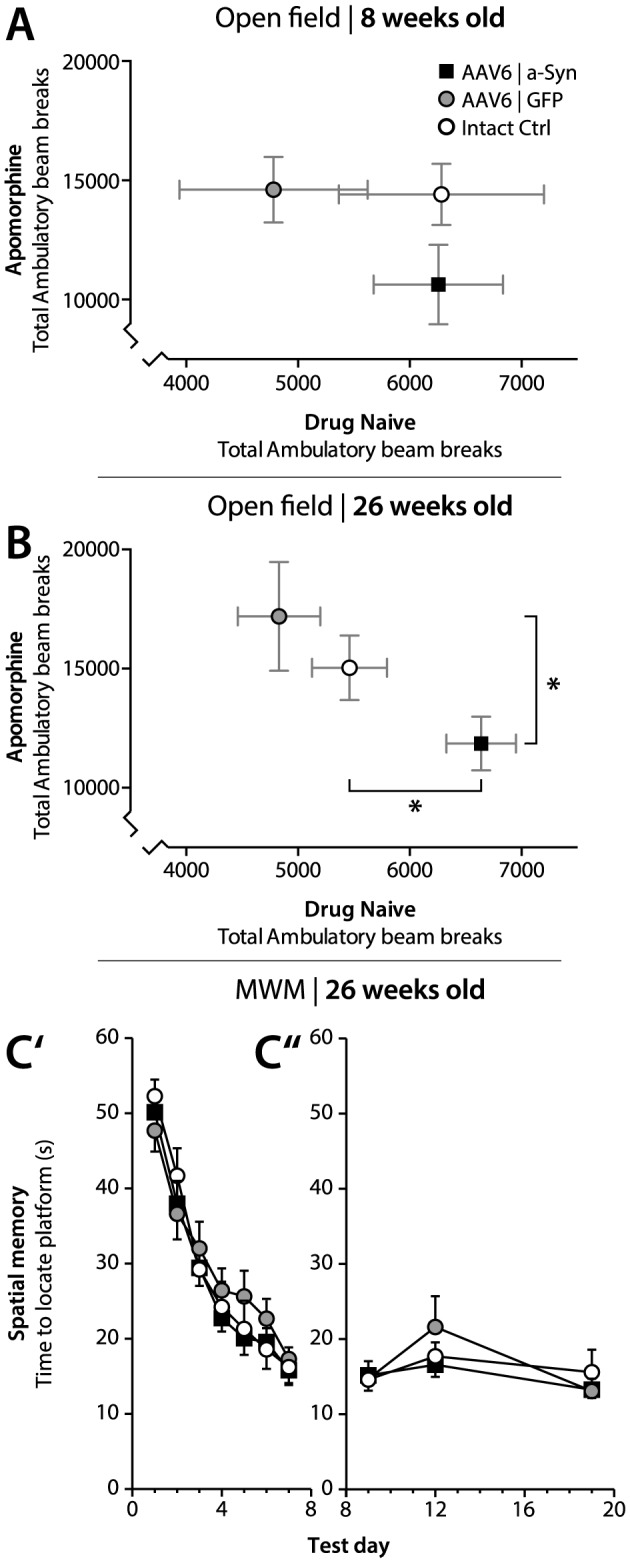
Behavioral assessment of motor and memory functions. At 8 weeks post viral injection, animals in the group receiving the AAV-6 α-synuclein vector (AAV6|a-Syn) displayed no significant difference in either naïve locomotion, or after subcutaneous apomorphine injection (1 mg/kg) relative to control groups (Intact Ctrl) or animals receiving the control vector (AAV6|GFP), in the open field task (A). However, at 26 wks post viral injection, the AAV6|a-Syn animals displayed a significant increase in naïve locomotion relative to control groups. In addition, the AAV6|a-Syn animals displayed significantly decreased response to apomorphine (1 mg/kg) relative to AAV6|GFP animals, suggesting dysregulation of the dopamine system (B). AAV6|a-Syn animals, trained in the Morris water maze (MWM) for seven consecutive days, exhibited no significant deficits in visio-spatial memory and learning (C′) and were also able to retain the learned task up to 21 days after the initial training period (C″). (* = p<0.05, one-way ANOVA with Newman-Keuls post hoc test). Data is expressed as mean ± SEM.

AAV6|a-Syn animals displayed no significant difference in exploratory behavior in the open field task, relative to control groups at 8 weeks post viral injection (Fig. 1A). However, at six months post viral injection, AAV6|a-Syn animals showed significantly increased naive exploratory behavior relative to both the AAV6|GFP (p = 0.01) and the intact Ctrl (p = 0.02) groups, indicating abnormal locomotor behavior (Fig. 1B). AAV6|GFP animals on the other hand, exhibited decreased ambulatory locomotion relative to both AAV6|a-Syn and intact Ctrl animals, indicating reduced exploratory behavior (Fig. 1B).

To investigate forebrain dopaminergic neurotransmission, animals were injected with the dopaminergic agonist apomorphine (1 mg/kg s.c.) and re-evaluated in the open field task. AAV6|a-Syn animals displayed a significantly lower locomotor activity relative to AAV6|GFP (p<0.05) with a strong trend relative to intact Ctrls, indicating a desensitization of dopaminergic striatal receptors or loss of postsynaptic neuronal targets at 6 months with indications of dysregulation already at 8 weeks (Fig. 1 A–B).

The cerebral cortices are important for many broad cognitive functions such as memory consolidation and retrieval and degeneration of cortical neurons in combination with the loss of cholinergic pathways is thought to contribute to dementia in DLB patients. To assess the role of α-synuclein over-expression on learning and memory formation rats were evaluated in Morris Water maze (MWM) task. Rats over-expressing α-synuclein displayed no learning deficits in the MWM task (Fig. 1C) and no significant difference between AAV6|a-Syn animals and control groups was observed in consecutive trials during the same trial day, indicating that the hippocampal dependent spatial working memory remained intact. We also evaluated the average swim speed of the animals to evaluate if the motor dysfunction observed in tests above could have been a confounding factor in this test. No differences were observed in the swim speed between the groups.

### Neonatal AAV6 injection induces widespread α-synuclein expression throughout the forebrain

**Figure 2:**
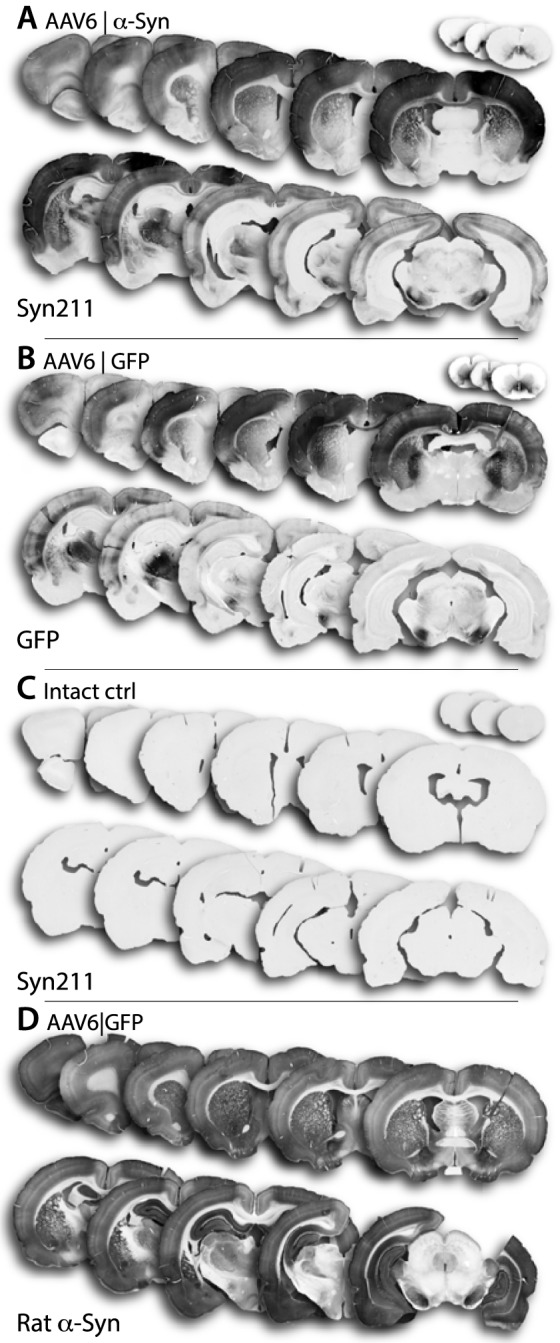
Histological overview of transduced brain regions. At 40 weeks post AAV injection, the long-term group was sacrificed and coronal brain sections stained with antibodies recognizing the respective transgene, syn211 against human α-Synuclein and a chicken polyclonal antibody against GFP. Intact control animals (Intact ctrl) were stained with both antibodies to confirm specificity (only syn211 shown). Transduction pattern was very similar in both the AAV6|a-Syn and the AAV6|GFP group with a very intense immunoreactivity in the cerebral cortex (A,B). Very low staining was observed in the Intact ctrl animals with the syn211 antibody (C), confirming the specificity of this antibody towards human α-synuclein. The expression pattern of human α-Syn (A) differed substantially from the expression pattern of endogenous rat α-synuclein as observer through staining with another antibody recognizing also the rat α-synuclein (BD Biosciences 610786) (D). For clarity, sections from the spinal cord are not to scale with the brain sections and have been enlarged to 200%.

The novel paradigm of neonatal viral delivery, using the AAV6 serotype, induced substantial expression of human α-synuclein throughout the forebrain, mainly localized to the cerebral cortices and striatum, visualized using the Syn211 antibody, previously shown to be selective towards human α-synuclein [26] (Fig. 2A). GFP expression from the control vector closely matched that of human α-synuclein (Fig. 2B), suggesting similar distribution and spread. Neurons positive for human α-synuclein were found to be widely dispersed throughout all cortical layers and in both projection and interneurons. Topographically, the main expression of human α-synuclein reached the highest levels in the M1/M2 motor cortices, with lower expression apparent in the pre- and infra-limbic frontal cortical areas. In addition, immunostaining revealed major anterograde transport of human α-synuclein, through the cortico-thalamic and cortico-spinal tracts, continuing through the pyramids of the medulla oblongata and persisting through the cortico-spinal tract along the ventro-medial spinal cord (Fig. 2A). Staining for human α-synuclein was also observed to extend along the striato-nigral pathway to the globus pallidus and the substantia nigra pars reticulata (SNpr), indicating substantial anterograde transport. Retrograde transport of human α-synuclein to the dopamine neurons in the SNpc was, however, not readily apparent. Large numbers of α-synuclein positive interneurons were also apparent within the olfactory bulb, suggesting efficient transduction of neuronal progenitor cells within the rostral migratory stream (RMS).

### Human α-synuclein gene expression and protein levels are increased throughout the forebrain

**Figure 3:**
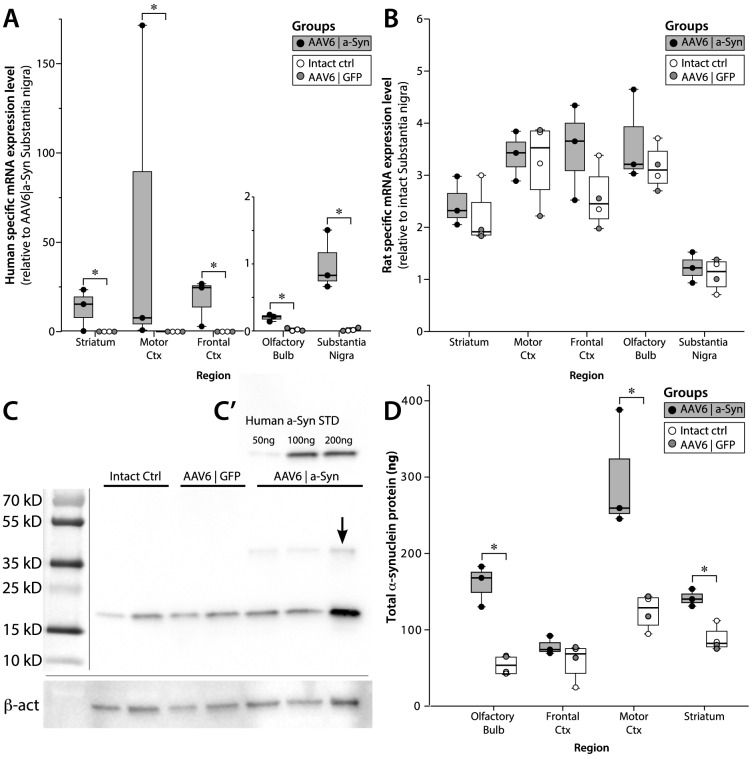
Molecular and biochemical quantification of α-synuclein expression. At 40 weeks post AAV injection, a separate group of animals was sacrificed for molecular and biochemical assessments. Fresh brain tissue was rapidly dissected into five regions where α-synuclein expression had been observed in the histological observations (Figure 1); striatum, motor cortex, frontal cortex, olfactory bulb and substantia nigra. Using trizol, mRNA and protein were extracted from each region and assessed using quantitative RT-PCR (A–B) and western blot respectively (C–D). For qPCR, two new primer pairs were designed, one specific for human α-synuclein (A) and one pair for rat α-synuclein (B). Both displayed very high specificity without cross-amplification >47000∶1). Human α-synuclein mRNA was robustly expressed in all regions tested in the AAV6|a-Syn animals without any detection in the control groups. Interestingly α-synuclein mRNA was also detected in the olfactory bulb, further confirming the observation that neuroblasts in the RMS were transduced in the neonatal brain. No changes were observed in the mRNA expression of endogenous α-synuclein (B). All data in A and B are normalized to three housekeeping genes (Gap-DH, TBP and β-actin). Total α-synuclein protein levels were quantified using quantitative western blot (C–D). The known amounts of human monomeric α-synuclein was loaded on each blot to create a standard curve (C′) and total α-synuclein was detected using an antibody previously shown to have comparable affinity to rodent and human α-synuclein in WB. Final expression levels were finally normalized to β-actin. Blot in (C) shows a representative blot of tissue from the motor cortex. Interestingly, despite samples boiled in SDS and run on SDS containing gels, samples from the AAV6|a-Syn animals displayed a immunoreactive band at exactly double the size of monomeric α-synuclein (arrow in C), an observation seen in all regions. This correlated with intensity of monomeric expression and was never found in any of the control animals. Total α-synuclein levels were increased up to four-fold in regions of the cortex near the injection site (D) with lower, although still significant, increase in the frontal cortex. (* = significant increase over control groups p<0.05 in Mann–Whitney U test). Data is expressed as mean ± SEM.

To evaluate the gene and protein levels of human α-synuclein, several regions of the forebrain and midbrain were dissected at 40 weeks post-injection and evaluated by qPCR and Western blotting. To allow for quantification of human α-synuclein and rat α-synuclein mRNAs independently, primers specific for human α-synuclein were developed and validated. Using these primers on both human and rat α-synuclein cDNA, we found a SNR between 47000∶1 and 112500∶1, *i.e.*, showing sufficient specificity. qPCR analysis revealed significant amounts of human α-synuclein mRNA levels in the striatum (p = 0.034), frontal (p = 0.034) and motor cortices (p = 0.034). Interestingly, significant levels of human α-synuclein were also detected in the olfactory bulb (p = 0.034) and ventral midbrain (most likely in axon terminals from medium spiny neurons projecting to the SNpr) (p = 0.034), although to a much lesser degree (Fig. 3A). To investigate if expression of human α-synuclein promotes any regulatory effect on mRNA levels of endogenous rat α-synuclein, primers specific for rat α-synuclein were used. qPCR analysis revealed no significant differences in rat α-synuclein between the treatment groups in any of the brain regions investigated, suggesting that over-expression of human α-synuclein does not regulate expression of endogenous rat α-synuclein (Fig. 3B).

Total rat and human α-synuclein protein expression was evaluated using Western blotting with monomeric α-synuclein protein as a standard detected with an antibody previously shown to display comparable affinity to rat and human α-synuclein [27] (Fig. 3C). AAV6|a-Syn animals showed a significant, 2–3 fold increase of total α-synuclein levels within the olfactory bulb (p = 0.034), striatum (p = 0.034) and the cerebral motor cortices (p = 0.034) were the expression levels of α-synuclein were found to be the highest (Fig. 3D). Interestingly, dimeric α-synuclein was present in all AAV6|a-Syn animals, even though proteins were extracted using SDS and run on a non-native gel, suggesting that some insoluble α-synuclein aggregates may have formed in the AAV6|a-Syn animals (Fig. 3C).

### α-Synuclein induces broad pathologies and increased phosphorylation throughout the forebrain

**Figure 4:**
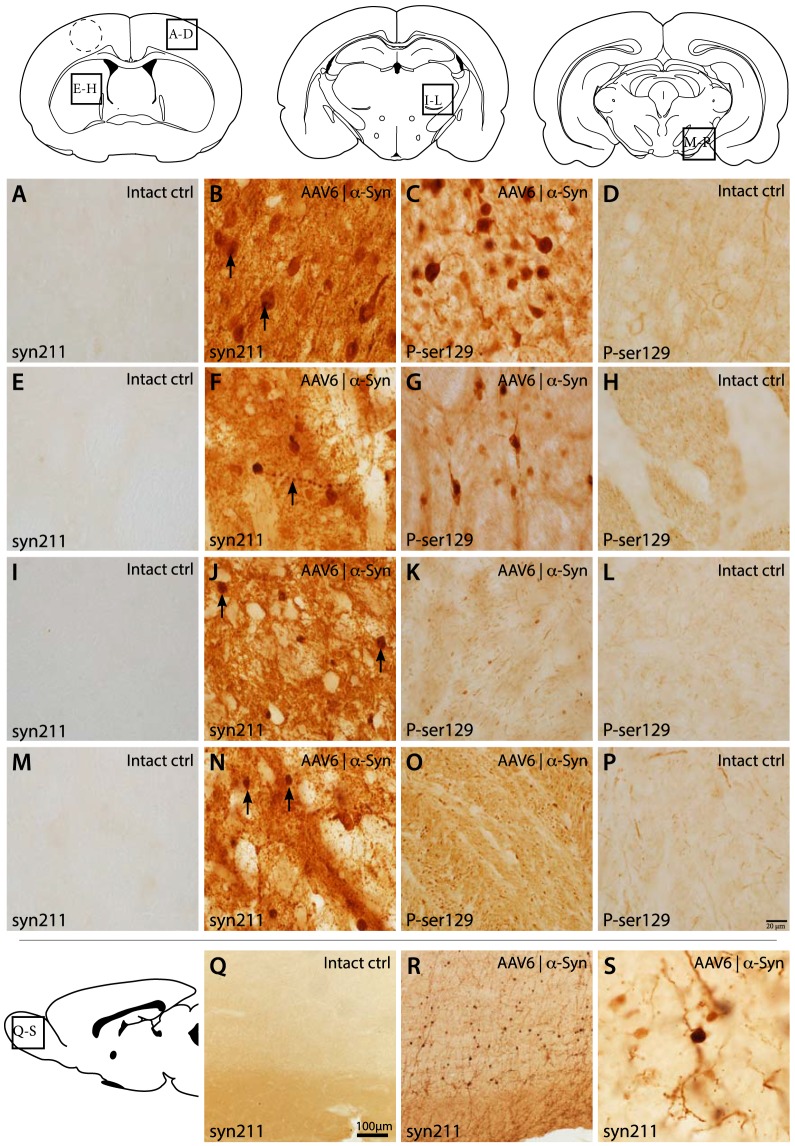
High-power histological analysis of α-synuclein expression. Histological analysis of the cerebral cortices revealed high expression of human α-synuclein (syn211) within the soma and axonal projections of the cortical neurons. These neurons displayed a high incidence of α-synuclein punctates within their soma (arrows), while phosphorylated α-synuclein (P-ser129) was highly increased within the neuronal soma, and to a lesser extent within the axonal projections (A–D). Striatal analysis revealed high incidence of neurons with prominent expression of human α-synuclein, with Lewy neurite's being a common find (arrow). Phosphorylation of α-synuclein within the striatal neurons was also upregulated (E–H) Projections within the thalamic nuclei were found to contain high levels of human α-synuclein with large α-synuclein positive structures, believed to be large axonal swellings, were present throughout the thalamus (arrows) (I–L). Large axonal swellings were also present to a large extent in the SNpR (arrows) while phosphorylation of α-synuclein appeared as only moderately increased (M–P). The neonatal injection of the AAV6 serotype displays an interesting ability to also infect neuronal progenitors within the rostral migratory stream (RMS) or the sub-ventricular zone. This is seen as mature neurons within the olfactory bulb expressing high levels of human α-synuclein (R–S). These neurons appear with healthy morphology with α-synuclein distributed throw-out the neuropil (S). Dotted lines within the cerebral cortex denote the position of the tissue punch taken for TEM analysis.

IHC staining revealed large numbers of neurons within the cerebral cortices and striatum expressing human α-synuclein. Neuronal cells displayed diffuse human α-synuclein staining throughout the soma accompanied by aggregate pathologies ranging from small α-synuclein positive puncta to larger inclusions (Fig. 4B), as well as large α-synuclein positive axonal swellings that were widespread throughout the cerebral cortices and the striatum (Fig. 4 A–H). Alpha-synuclein staining within the thalamic nuclei and SNpr revealed abundant α-synuclein positive projections but no transduced cell bodies (Fig. 4 I–P). Large pathologies with intense α-synuclein staining in both the thalamic nuclei and the SNpr were thought to be exceptionally large axonal swellings, created as a result of blocked anterograde transport and α-synuclein accumulation (see EM data below).

**Figure 5:**
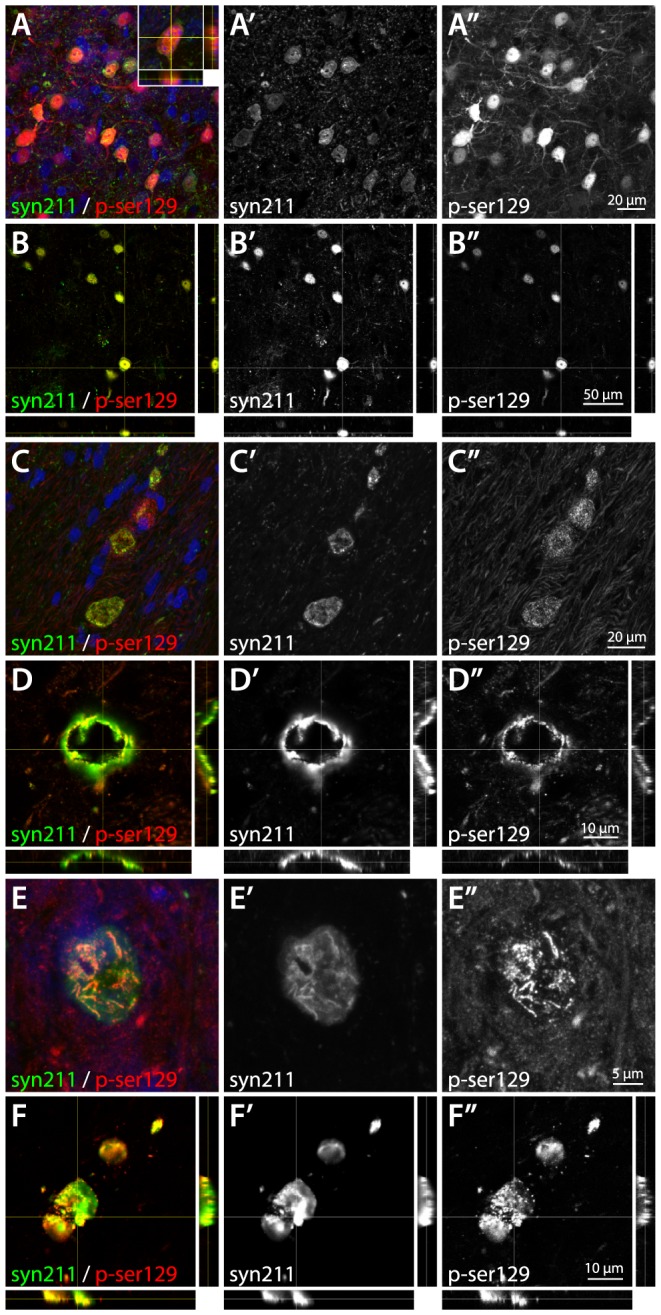
Analysis of cortical pathologies in AAV6|a-Syn animals using double fluorescence confocal microscopy. Human α-synuclein was found to be localized both in the nucleus and the cytosol of transduced neurons (A–B″). In cells with lower expression level, expression was mostly confined to the cytosol (A′), while with increase of the expression, nuclear localization increased substantially (B′). Interestingly, α-synuclein localized to the nucleus appeared to be relatively more phosphorylated than cytosolic α-synuclein (inset in A, A″ and B″). Phosphorylated human α-synuclein also appeared in larger inclusion bodies outside the nucleus. These appeared both as large hollow spheres (C–D″), assemblies of fibril-shaped protein (E–E″) and flat sheets (F–F″). In all these cases, the majority of the aggregated protein appeared to be phosphorylated at serine 129 (C″, D″, D″ and F″). If these inclusion bodies are localized inside or outside the cell membrane cannot be conclusively determined at this point. Images in panels A, C and E are displayed as maximum intensity projections and B, D and F as orthogonal projections. All captured using sequential laser scanning at Airy 1 pinhole.

Phosphorylation of α-synuclein, at serine 129, (pSer-129) is thought to be a post-transcriptional modification associated with α-synuclein aggregation and pathology. Staining for pSer-129 α-synuclein revealed a significant increase of phosphorylation throughout the cerebral cortical layers and striatum. Phosphorylation of α-synuclein was mainly found within the neuronal cell soma and, to a lesser extent, in cortical and striatal projections (Fig. 4 C and K). Moderately increased phosphorylation was apparent in thalamic nuclei and the SNpr, although to a much lesser extent than within the cerebral cortices and the striatum and was notably different to the Syn211 human α-synuclein staining pattern. These findings suggest that phosphorylation takes place mainly in the cell soma and may be less prone to anterograde transport, relative to non-phosphorylated α-synuclein *in vivo*. Phosphorylated α-synuclein appeared to co-express with human α-synuclein in the neuronal cell soma (Fig. 5 A–B), mainly as intracellular puncta and within larger inclusion bodies (Fig. 5 C–F), suggesting that phosphorylated α-synuclein may be more prone to aggregation *in vivo*. Hyper-phosphorylation of AD associated Tau protein, may be present simultaneously in patients with DLB and AD was also evaluated by immunohistochemistry. However, no Tau protein hyper-phosphorylated at the pSer396 residue could be identified in any of the brain regions affected by α-synuclein over-expression (data not shown).

### Human α-synuclein localizes to cell soma, cell nuclei, axonal swellings and synapses, and is correlated with neuronal pathology

**Figure 6:**
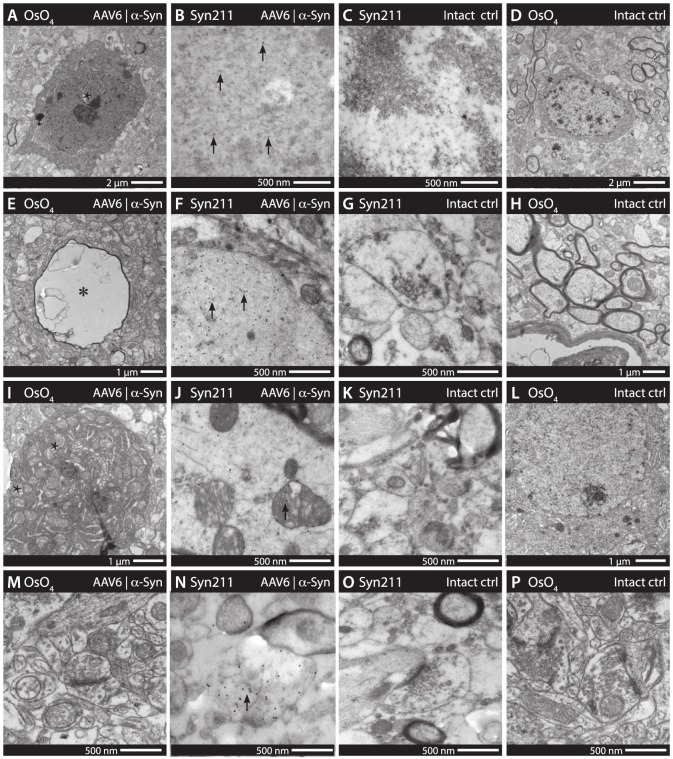
Transmission electron microscopy assessing α-synuclein localization and associated pathologies. Analysis of the cerebral cortices through electron microscopy revealed a large number of apoptotic neurons. These neurons were identified by morphological features, such as large, swollen mitochondria, vacuole formation and disruption of the nuclear envelope (*e.g.*, * in A). Immunogold staining for human α-synuclein (Syn211) revealed a high presence of nuclear associated α-synculein in apoptotic neurons (black 10 nm dots marked by arrows in B) in addition to that of the soma (A–D). Large axonal swellings were also a common pathological finding throughout the cerebral cortices (* in C). These were found to have large accumulations of human α-synuclein (arrows in F) and also appeared to associate with the cellular membrane (E–H). Large and swollen mitochondria present in neuronal cells, as well as within the axons (* in I) appeared associated with large amounts of human α-synuclein (I–L). Human α-synuclein was also found to be present to a large extent in synapses of the cerebral cortices (M–P), However, these synapses appeared largely of normal morphology. The antibody staining in B–C, F–G, J–K and N–O is visualized using immunogold particles of 10 nm in diameter.

**Figure 7:**
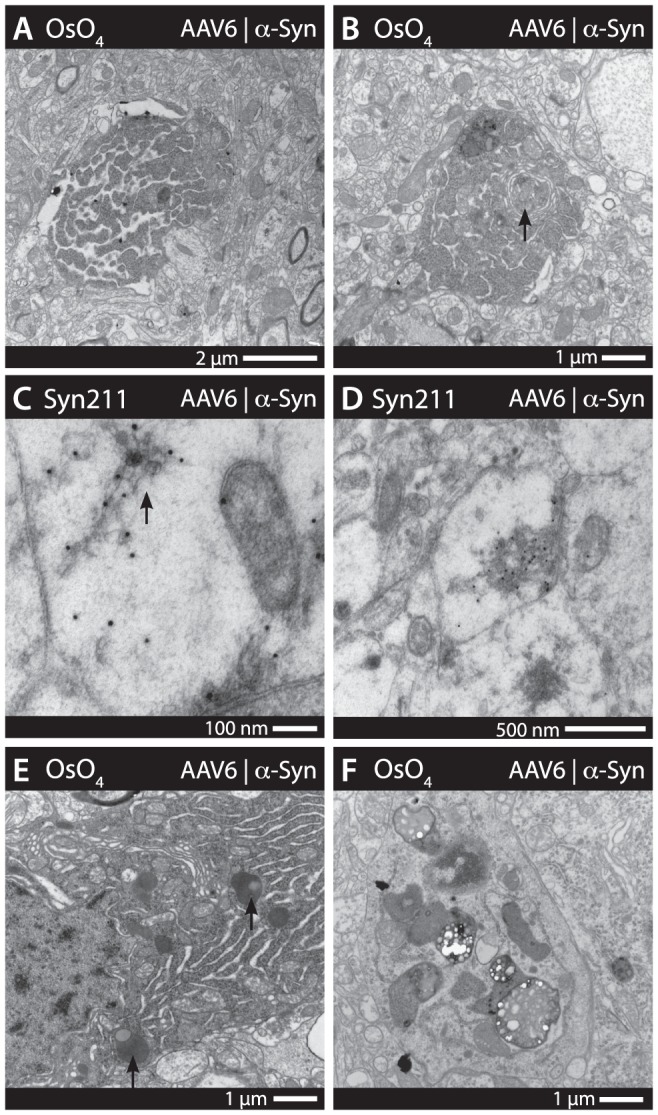
Immunogold and osmium developed TEM reveals α-synuclein associated, intracellular fibrillar structures and increase of autophagosomes. Additional electron microscopy revealed a number of pathological features, including apoptotic body cell remnants, present throughout the cerebral cortices (A). Autophagosomes forming from the Golgi (arrow) was a feature also present in cells with a high degree of apoptotic morphology (B). Human α-synuclein immunogold (syn211) staining revealed fibrillar-like structures (arrow in C) as well as larger inclusion bodies (D) present within the soma and axons (C–D). Subjectively, granular aggregates of lipofucins appeared to be a more common feature in α-synuclein expressing animals, suggesting lysosomal/proteosomal pathology. However, no absolute quantification was possible. (E–F).

Electron microscopy on Osmium tetroxide (OsO4) treated brain sections revealed a large number of cells with apoptotic morphology, characterized by fragmentation of the nuclear membrane, distended rough endoplasmic reticulum and swollen mitochondria (Fig. 6). Apoptotic cell morphology ranged from early apoptotic features to small, condensed cell fragments. In some cases, autophagosomes derived from the Golgi were also identified within highly apoptotic cells (Fig. 7 A–B). Using immunogold staining against human α-synuclein (Syn211), apoptotic cells presented with large amounts of nuclear associated α-synuclein. The α-synuclein found in the nuclei appeared mainly associated with areas of chromatin (Fig. 6 B), supporting previous findings that α-synuclein may associate with DNA-histone complexes and promote neurotoxicity. Diffuse staining of human α-synuclein was observed in numerous cells, however, α-synuclein was also present as larger inclusion bodies and fibrillar structures (Fig. 7 C–D), some within vesicle structures that may be part of the lysosomal system (Fig. 7 E–F). Cells with degenerative morphology were often found to contain large numbers of large, swollen mitochondria (Fig. 6 I–J). Human α-synuclein was found to be associated with such pathological mitochondria (Fig. 6 I–J), as well as to mitochondria without any clear pathological morphology. Axonal swellings were also a relatively common finding in animals expressing human α-synuclein. These swellings appeared as large, empty structures, devoid of characteristic microtubules (Fig. 6 E). In sections stained for human α-synuclein, these large axonal swellings were found to be full of human α-synuclein, suggesting α-synuclein driven pathology (Fig. 6 F). Finally, large amounts of human α-synuclein was also found within synapses, although no morphological pathologies were apparent (Fig. 6 M–N).

### AAV-mediated α-synuclein over-expression causes cortical neurodegeneration

**Figure 8:**
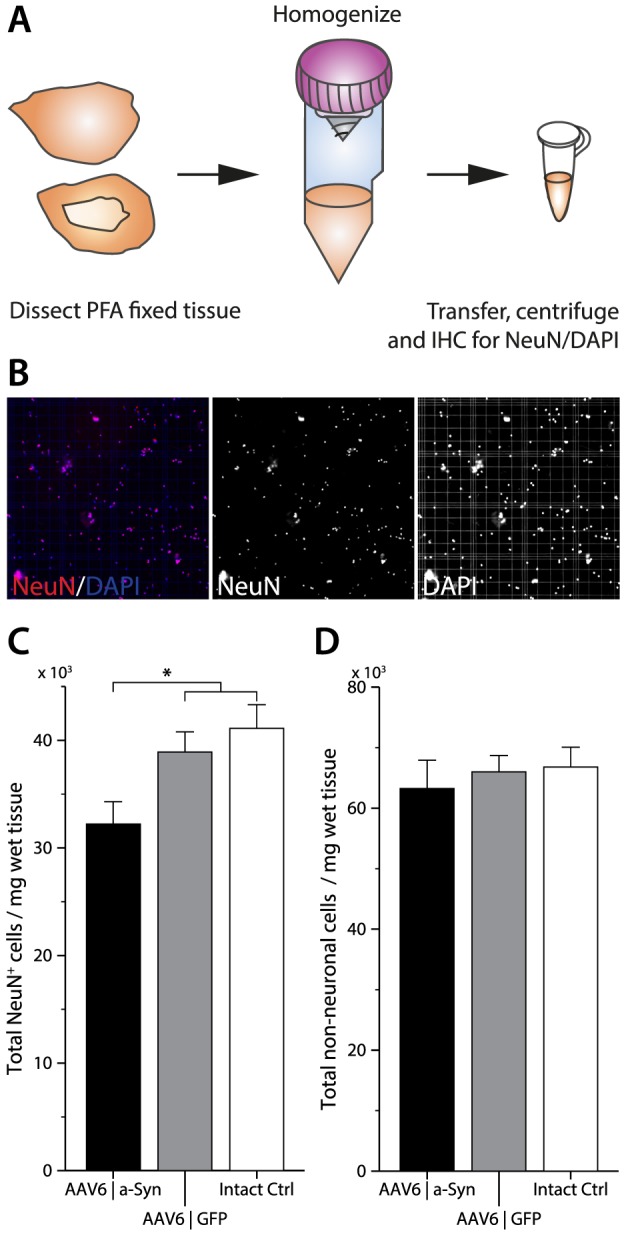
Modified isotropic fractionation for neuronal quantification reveals broad-spread cortical degeneration. Principals of modified isotropic fractionation for neuronal quantification (A). PFA fixed cerebral cortices were dissected and dissociated using the GentleMacs tissue dissociator. Following centrifugation and washing only a nuclear fraction remained. The nuclear fraction was stained for DAPI and the NeuN neuronal marker and counted in a haemocytometer, using florescence microscopy (B). Quantification revealed a significant neuronal degeneration within the cerebral cortices of animals expressing α-synuclein, relative to controls (C). There was however, no significant neuronal loss in the cerebellum, a region not expressing human α-synuclein, using our modified isotropic fractionation protocol (AAV6|a-Syn 463.1±24.9×10^3^, AAV6|GFP 475.1±15.1×10^3^, Intact ctrl 506.7±12.1×10^3^. (* = p<0.05, one-way ANOVA with Newman-Keuls post hoc test). Data is expressed as mean ± SEM.

Quantification of forebrain neurons is essential to evaluate α-synuclein induced toxicity that may lead to neuronal degeneration. Thus, a modified isotropic fractionation protocol for neuronal and non-neuronal quantification was developed. Assessing such a large and heterogeneous brain region is difficult and time consuming using established stereological methods, which require frequent sampling of homogeneous regions to be accurate. Isotropic fractionation, on the other hand, allows cell quantification and accurate comparison between samples, regardless of differences in volume [21], [28]. Therefore, we used a modified protocol based on the isotropic fractionation method to obtain quick and accurate quantification of neurons throughout the forebrain (Fig. 8 A–B). Intact control animals displayed a mean total of around 43000 NeuN positive cells/mg tissue. AAV6|a-Syn animals, killed 40 weeks after vector injection, differed significantly (p = 0.02), with an average of 34000 NeuN positive cells/mg tissue, from that seen in intact control and GFP expressing animals (Fig. 8 C). Control animals had a mean total value of around 67000 DAPI positive cells/mg tissue, while total cells in animals expressing human α-synuclein displayed a non-significant reduction in mean total cells of around 63000 DAPI positive cells/mg tissue (Fig. 8 D). Our novel isotropic fractionation protocol was evaluated and validated by quantifying total cell and total neuronal populations in the cerebellum in all groups, as the cerebellum, a structure located outside the vector-transduced parts of the brain. No significant difference in the number of total DAPI positive and NeuN positive cells was found between the animals expressing human α-synuclein and the two control groups.

### α-Synuclein over-expression induces progressive neuronal degeneration and loss of cholinergic cortical and striatal interneurons

**Figure 9:**
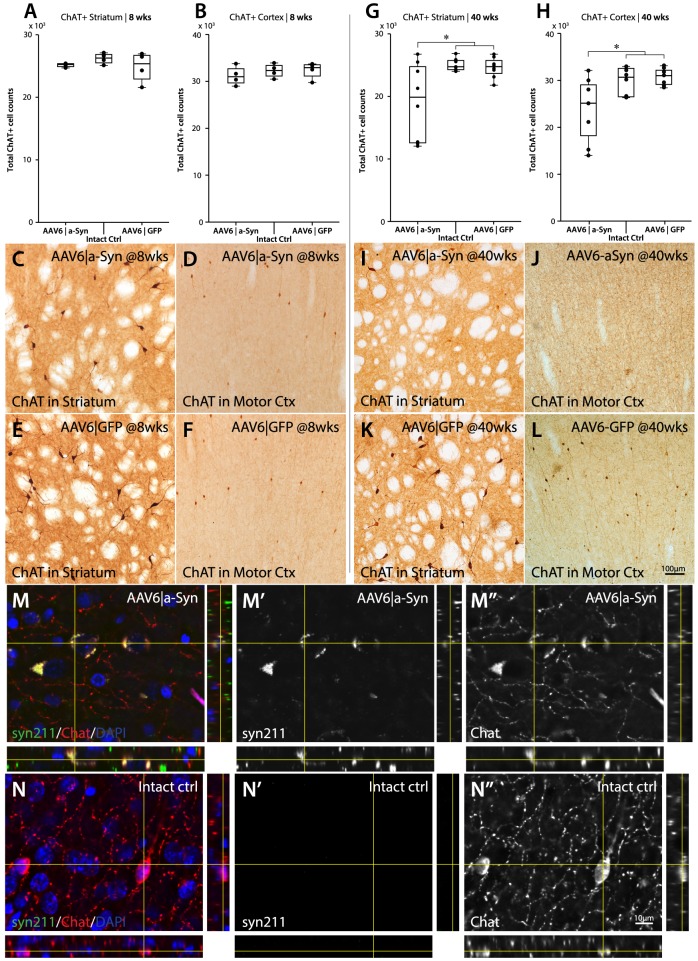
Quantification of cholinergic neurons regions with high expression of human α-synuclein. Immunohistochemical staining of ChAT positive Cholinergic interneurons using DAB precipitation coloring of the striatum and cerebral cortex revealed a significant, progressive loss in both the striatum and the medial lateral Cerebral cortices starting later than 8(8 wks) no loss of cholinergic neurons was apparent in either the striatum (A) or the cerebral cortices (B). However, ChAT + interneurons were qualitatively lighter stained with fewer processes clearly visible (C–D) relative to GFP injected animals (E–F). At 10 months post viral injection, however, there was a significant reduction on Cholinergic interneurons in both Striatum (G) and Cortex (H) of AAV6|a-Syn animals compared to both AAV6|GFP and Intact ctrl animals. At this time point, ChAT staining revealed a high degree of clearance of cholinergic neurons within the striatum (I) and medial lateral cerebral cortices (J), relative to GFP injected animals (K–L). (* = p<0.05, one-way ANOVA with Newman-Keuls post hoc test). Data is expressed as mean ± SEM.

Reduction in cortical acetylcholine levels has been reported to play a major role in the cognitive deficits observed in DLB patients. To evaluate the loss of cholinergic neurons in the cortex due to α-synuclein over-expression, stereological analysis of ChAT positive neurons in the dorso-medial cerebral cortices was performed on animals 10 months post-injection. The stereological analysis revealed a significant neuronal loss (p<0.05) of ChAT positive cholinergic neurons within the cerebral cortices, relative to GFP and intact controls, suggesting α-synuclein specific toxicity (Fig. 9 H, J). In order to assess if the observed neuronal degeneration was progressive or was a result of an earlier, acute degeneration in response to α-synuclein over-expression, animals expressing human α-synuclein were evaluated 8 weeks post-injection. At this time-point, no neuronal degeneration in cortical neuronal ChAT+ cells could be observed, suggesting a progressive neuronal loss (Fig. 9 B,D). As α-synuclein over-expression is also present in the striatum and the cholinergic interneurons there may be part of the dysregulatory response to apomorphine in the open field task, stereological analysis of the striatal cholinergic interneurons was also performed. Animals expressing human α-synuclein displayed a significant neuronal loss of cholinergic interneurons at 10 months post viral injection (Fig. 9 G,I), while this was not observed in animals at 8 weeks post viral injection (Fig. 9 A,C), again, suggesting a progressive neuronal loss.

### α-Synuclein over-expression, induces activation and promotes pathological morphology in resident microglia

**Figure 10:**
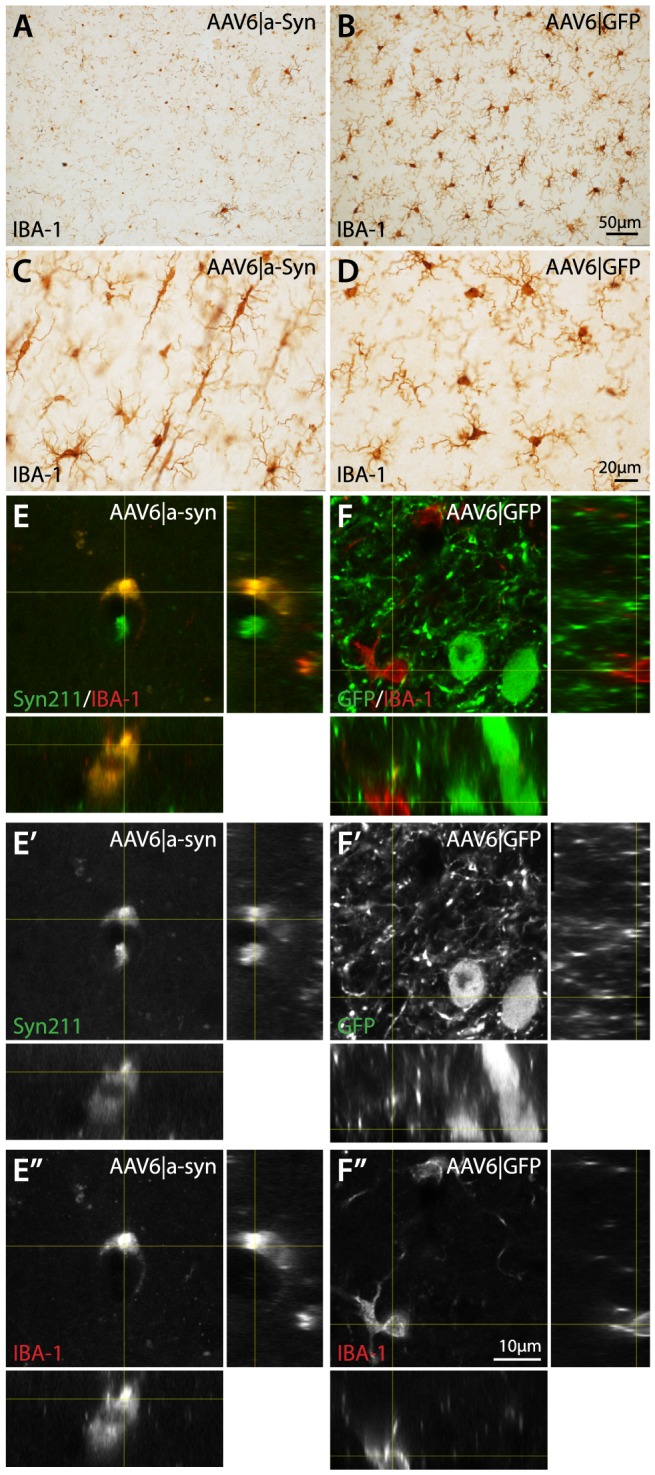
Morphological changes and evidence of α-synuclein uptake in IBA-1 positive microglia. Immunohistochemical staining of IBA-1 using DAB precipitation coloring in the cerebral cortex revealed a substantial change in the microglial morphology in the cortex of AAV6|a-Syn animals compared to AAV6|GFP and Intact ctrl animals at 10 months post AAV injection (A–D). In the regions overlapping with the most intense human α-synuclein over-expression, most microglial cells appeared with very few visible processes and majority of the IBA-1 positive staining localized to the nucleus (A), this stood in stark contrast to the normal IBA-1 staining of microglia in the corresponding cortical regions of AAV6|GFP animals (B). In surrounding cortical regions, the microglial morphology in AAV6|a-Syn animals showed a second distinct morphology, with elongated cell body and fewer processer, suggesting a migratory profile (C). Using double-labeled, fluorescence with IBA-1 and human α-synuclein/GFP antibodies in a scanning confocal microscope, the presence of α-synuclein in microglia was investigated (E–F″). At the core of the human α-synuclein over-expression in the AAV6|a-Syn animals, microglia were found with significantly changes morphology and intra-cellular α-synuclein (E–E″). Interestingly, two localization patterns were observed, both in the cell soma in circular, vesicular structures and as small accumulations in distal processes of the microglia. No such patterns were observed in AAV6|GFP (F–F″) or intact controls.

Microglial activation and reactivity has been found to be increased in the brain of DLB patients [29] and may contribute to disease progression. In our animal model, over-expression of human α-synuclein was restricted to neurons through the utilization of the neuron-specific human Synapsin-1 promoter [22]. Nevertheless, α-synuclein over-expression caused distinct phenotypic changes in microglial morphology. First, a large fraction of microglial cells were found with highly condensed cytoplasm and nuclei, with a loss of extending processes (Fig. 10 A), indicating increased activity and reactivity in response to α-synuclein over-expression. The second change was observed as a general elongation of the microglial cytoplasm coupled with loss of extending processes, indicating increased activity and migration (Fig. 10 C). These morphological changes were readily identified within the main transduction area of the cerebral cortices but not in proximity to α-synuclein positive projections in other brain regions. The morphological changes were also not observed in AAV6|GFP or intact control animals (Fig. 10 B, D). To investigate if the morphological changes in the microglial cells could be a result of direct uptake of human α-synuclein, tissue sections were examined using confocal microscopy (Fig. 10 E–F). Human α-synuclein was found within microglial cells from AAV6|a-Syn animals and was present mainly as intracellular inclusion bodies and within IBA-1 positive projections throughout the cerebral cortices (Fig. 10 E). These cells were mostly found in close proximity to human α-synuclein positive neurons displaying degenerative morphology. In addition, some microglia were observed to contain large vesicular structures loaded with α-synuclein (Fig. 10 E), indicative of an active clearance mechanism. Taken together, these data suggest that microglial uptake of human α-synuclein participates in the ongoing pathology in this animal model.

## Discussion

In this study we set out to develop a novel rat model of widespread progressive forebrain synucleinopathy. The goal was to achieve pathology restricted to this region without affecting other parts of the brain, such as hippocampus and the substantia nigra. Rodent models of diverse forms of synucleinopathy, such as PD, and DLB, have been valuable in determining both vulnerability factors and the specific contribution of α-synuclein-induced pathology to the phenotype [30]–[34]. In α-synuclein overexpressing transgenic mice, however, the specific contribution of pathology induced in different parts of the brain has been difficult to achieve, since most of these transgenic mouse strains express the protein in many parts of the brain [11], [35], [36].

Through the neonatal AAV6-mediated gene delivery paradigm together with the injection coordinates utilized in this experiment, α-synuclein expression was mainly expressed within neurons in the M1–M2 regions and to a lesser extent also in the pre- and infra-limbic prefrontal cortex and the striatum. The mechanism behind the prominent cortical transduction following AAV injection into the striatum of neonatal rats is currently unknown. It is clear that AAV particles diffuse much more efficiently in the neonatal brain, compared that that in adults [18], [19]. This is not only due to the smaller size of the brain in neonates but most probably also to the absence of myelinated structures that are known to restrict the diffusion of the viral particles from the injection site. In preparation for this experiment we compared the usefulness of two different AAV serotypes, AAV5, which has been used in previous studies [18], [19], and AAV6, which is our preferred serotype. We found that AAV5 induced broad expression of human α-synuclein throughout the forebrain but did not display the same high affinity for neurons in the neocortex as the AAV6 vector. Thus, the properties of the AAV6 capsid are particularly favorable for targeting neurons in the neocortex. The broad transduction pattern is not believed to be caused by a leakage of the vector into the CSF, as neurons in the hippocampus and other structures surrounding the caudal parts of the ventricles are not transduced. We have also conducted pilot studies injecting the AAV6 vector directly into the ventricles and the resulting transduction pattern is then restricted mainly to the ependymal cells with very little parenchymal penetration. The pattern of α-synuclein expression in the olfactory bulb, confined to a subset of interneurons, suggest that the AAV6 vector is efficient in transducing the neural progenitors in the subventricular zone and/or the RMS. This labeling, which was not observed with the AAV5 vector, further supports the beneficial permissiveness of AAV6 to immature and still dividing neural progenitors. Postnatal cell division in the cerebral cortex [28] may therefore also contribute to the efficient cortical transduction obtained with the AAV6 vector. α-Synuclein expression did not extend into the hippocampus or other parts of the temporal lobe. The absence of synucleinopathy in this region is consistent with the absence of spatio-visual memory deficits in the MWM, a test known to be highly hippocampus dependent [37].

In post-mortem analysis performed at 40 weeks post injection, *i.e.* when the rats had reached 9 months of age, the animals displayed a multifaceted degenerative pattern with a number of very interesting pathological features. Isotropic fractionation based quantification showed an overall loss of cortical neurons of more than 20% in the AAV6|a-Syn animals. Such a loss is impressive in the light of that the entire cerebral cortex was included in this counting and, as the expression of α-synuclein is primarily in the frontal parts, the degeneration in this region is most likely even greater. In addition, widespread α-synuclein –induced pathology was present in many surviving neurons, associated with widespread microglial activation. α-Synuclein was distributed diffusely throughout the neuronal cytoplasm and also as puncta and inclusion bodies of varying size in both the soma and the nucleus. Immunogold staining on EM sections revealed that α-synuclein formed fibrillar-like structures as well as larger inclusion bodies within the transduced neurons.

Phosphorylated α-synuclein was observed in the form of small puncta within larger inclusion bodies present in the cytoplasm, supporting the notion that phosphorylation is important for aggregation, though not absolutely required [38]. However, this still remains an open question and there are studies showing that S129 phosphorylation has a negative effect on aggregation [39]. Therefore, the phosphorylation may also have happened after the synuclein accumulated. It remains to be shown if the amounts of phosphorylation in this model follows the amount of human α-synuclein over-expression or if it is increased to an even larger extent, then suggesting a potential seeding effect of the human α-synuclein on the endogenous protein. Interestingly, phosphorylated α-synuclein was commonly associated with the cell nucleus [40], [41]. This is an important finding as nuclear localization may further aggravate neuronal toxicity [42]. Indeed, electron microscopy analysis revealed that neurons with large amounts of human α-synuclein present within the nucleus often presented with a degenerative morphology.

Human α-synuclein was observed to be anterogradely transported along the cortico-thalamic and cortico-spinal tracts, as well as in striatal projection neurons. This fits well with previous studies showing that α-synuclein plays a role in synaptic function [43]–[45]. Although found in some neuronal projections, phosphorylated α-synuclein was not as common there as non-phosphorylated α-synuclein, especially in longer projections, suggesting that phosphorylation impacts anterograde axonal transport of α-synuclein *in vivo*. This is in line with previous studies showing that phosphorylation of α-synuclein attenuate axonal transport in cultured neurons [46]. Human α-synuclein was a common finding in numerous axonal swellings, localized not only in the cortex but also within the striatum, thalamus and SNpr. The axonal swellings indicate that α-synuclein over-expression causes blockages or disruption of axonal trafficking, which would further contribute to pathology and neuronal degeneration [46], [47].

In this study we also chose to study one cellular subtype of interest in the normal function of the forebrain, the cholinergic interneurons. Cholinergic interneurons have recently received increased attention, as they are important for intrinsic regulation of a wide range of behaviors, including associative learning [48], reward processing [49], depression [50] and dyskinesias in PD [51]. Furthermore, a decrease in forebrain acetylcholine levels is observed in many DLB cases and pathological changes in cholinergic interneurons may be a contributor to this disease phenotype [52].

Stereological counting showed a progressive loss of ChAT+ interneurons, both in striatum and cortex of AAV6|a-Syn animals. While variable, we found up to a 50% reduction of ChAT+ interneurons both in the striatum and in the cortex. This is a proportionally greater reduction than we observed in the total loss of neurons in the cortex using the isotropic fractionation, indicating that cholinergic interneurons may be more vulnerable to α-synuclein toxicity than other sub-types of cortical neurons.

Consistent with the widespread α-synuclein-induced pathology the AAV6|a-Syn animals displayed significantly increased locomotor behavior in the open field task, linked to a decreased locomotor response to apomorphine. While there are many possible functional explanations for this particular phenotype, α-synuclein mediated pathology and loss of cholinergic interneurons in the striatum, provides one possible mechanism. Both cholinergic and GABAergic interneurons in the striatum play a major role in modulating output from the medium spiny neurons to the direct and indirect pathways [53], [54]. Cholinergic interneurons within the striatum work in close proximity with dopaminergic synapses and modulate dopamine release and the response of the medium spiny neurons to dopamine agonists [55]. It is also worth to note that while the degeneration of striatal cholinergic interneurons is not a prominent feature in either DLB or other forms of dementia, they have been shown to be affected in related disorders such as supranuclear palsy (PSP) [56].

The utilization of both morphologic EM and immunogold labeled EM in this study allowed us to study the α-synuclein-induced toxic mechanisms in both soma and axons of degenerating and dysfunctional neurons. The pathological morphology ranged from disruption of the nuclear membrane and distention of the endoplasmatic reticulum, to apoptosis with evidence of autophagy. Similar findings have recently been observed with mutations of β-synuclein linked to DLB [57]. Autophagy represents a major route for degradation of aggregated cellular proteins and dysfunctional organelles and has emerged as an interesting potential target for neuroprotective therapies, relevant for both α-synuclein- and tau-induced toxicity [58]–[61]. Immunogold staining also revealed that human α-synuclein had a strong association to mitochondria. These mitochondria often presented with a swollen pathological morphology, although this was not always the case. Indeed, mitochondrial dysfunction has been identified as one of the hallmarks of α-synuclein toxicity and is thought to play a major role in neuronal degeneration [58], [62].

Lastly, it is worth to stress that we through the neonatal injection paradigm achieve widespread protein injection and significant pathology without the requirement of exceptionally high α-synuclein expression levels. In areas of most prominent pathology, the total α-synuclein levels were increased to, on average, two-fold. This is very similar to the levels observed in patients with SNCA gene triplication (coding for α-synuclein) of the Swedish-American kindred, which also displays severe dementia, Parkinsonism and Lewy pathology both in cortex and in the hippocampus [63]. However, individual neurons within the transduced region may express relatively higher amounts. Furthermore, as we in this model restrict expression of human α-synuclein to the cerebrum, this opens up for peripheral studies of wet biomarkers of disease progression, *e.g.*, in CSF or blood, as any human α-synuclein observed there can be directly attributed to central expression.

### Concluding remarks

Viral vector gene delivery has shown great promise in development of novel models replicating human monogenic disease conditions in animals. Using this technique we have created a novel animal model, based on over-expression of human α-synuclein, that replicates the cortical neuronal degeneration and pathology seen in DLB patients, without the confounding contributions of midbrain and/or brainstem pathology. This study has for the first time shown that cholinergic interneurons in the cortex and striatum are selectively vulnerable to α-synuclein induced toxicity and that early, AAV-mediated α-synuclein delivery produces a progressive neurodegeneration with chronic microglial activation. The pathology in this model replicates a number of important findings in other *in vivo* models of α-synuclein mediated toxicity, such as nuclear localization of phosphorylated α-synuclein, swollen mitochondria with membrane associated α-synuclein, increased autophagy, and microglial activation with α-synuclein uptake. Therefore, this rat model could become very useful for studies of novel disease modifying therapies.

Although we used human wild-type α-synuclein in our study, the neonatal gene delivery approach described here may be used investigate other, mutated forms of α-synuclein, and it will be generally useful for studies involving widespread gene delivery to neurons in the neocortex. We also propose that our animal model may be used as a platform for discovering and evaluating novel biomarkers, such as development of diagnostic imaging tools like SPECT, PET and functional MRI. These may be used for evaluating hypoperfusion, hypometabolism, and global grey matter atrophy. Taken together, this model exhibiting α-synuclein over-expression localized to the cortices provides significant advantages over current transgenic models for evaluating the mechanisms observed in conditions of widespread cortical α-synuclein pathology, such as DLB.
